# A versatile genomic transgenesis platform with enhanced *λ* integrase for human Expi293F cells

**DOI:** 10.3389/fbioe.2023.1198465

**Published:** 2023-06-22

**Authors:** Asim Azhar Siddiqui, Sabrina Peter, Eve Zi Xian Ngoh, Cheng-I. Wang, Shirelle Ng, John A. Dangerfield, Walter H. Gunzburg, Peter Dröge, Harshyaa Makhija

**Affiliations:** ^1^ School of Biological Sciences, Nanyang Technological University, Singapore, Singapore; ^2^ Singapore Immunology Network, Agency for Science, Technology and Research (A*STAR), Singapore, Singapore; ^3^ Austrianova Singapore Pte. Ltd., Singapore, Singapore; ^4^ Department of Pathobiology, Institute of Virology, University of Veterinary Medicine, Vienna, Austria; ^5^ LambdaGen Pte. Ltd., Singapore, Singapore

**Keywords:** human cell line engineering, *λ* integrase, Expi293F cells, biotherapeutics, site-specific transgenesis, microencapsulation

## Abstract

Reliable cell-based platforms to test and/or produce biologics in a sustainable manner are important for the biotech industry. Utilizing enhanced λ integrase, a sequence-specific DNA recombinase, we developed a novel transgenesis platform involving a fully characterized single genomic locus as an artificial landing pad for transgene insertion in human Expi293F cells. Importantly, transgene instability and variation in expression were not observed in the absence of selection pressure, thus enabling reliable long-term biotherapeutics testing or production. The artificial landing pad for λ integrase can be targeted with multi-transgene constructs and offers future modularity involving additional genome manipulation tools to generate sequential or nearly seamless insertions. We demonstrated broad utility with expression constructs for anti PD-1 monoclonal antibodies and showed that the orientation of heavy and light chain transcription units profoundly affected antibody expression levels. In addition, we demonstrated encapsulation of our PD-1 platform cells into bio-compatible mini-bioreactors and the continued secretion of antibodies, thus providing a basis for future cell-based applications for more effective and affordable therapies.

## Introduction

Therapeutic products that are derived from living organisms are known as biotherapeutics and are the fastest-growing categories of products in the pharmaceutical industry including, but not limited to, monoclonal antibodies, signalling molecules and blood factors that are being produced in mammalian cell lines (Johnson, 2018; Walsh, 2018). Hence, development and manufacturing of biotherapeutics hinge on genetically stable producer and/or tester cells capable of producing recombinant proteins efficiently. Furthermore, emerging cell encapsulation technologies have enabled possible new applications for mammalian producer cells as mini-bioreactors for *in vivo* cell-based therapies (Ashimova et al., 2019; Nash et al., 2022b).

Mammalian cells have certain advantages over other expression systems such as those derived from bacterial, yeast or insect origin. They have the desired features to express large and complex proteins with proper folding and post translational modifications (Kim et al., 2012; Kuriakose et al., 2016). Chinese Hamster Ovary (CHO) cells, an immortalized epithelial cell line, are the current workhorse of the biopharmaceutical industry resistant to human pathogen infection (Lalonde and Durocher, 2017). Expi293F cells, derived from human embryonic kidney cells (HEK293) can provide an alternative to CHO cells. These cells can grow in suspension cultures at high density to produce high levels of proteins from episomal or chromosomal transgenes (Fang et al., 2017; Ecker et al., 2020). HEK293 cells have a significant history of use in the development of cell and gene therapy products (Ayuso, 2016; Dumont et al., 2016; Merten et al., 2016), and GMP-qualified HEK293/Expi293F cells are available (Salmons et al., 2007).

Multiple genome-editing tools like zinc finger nucleases (ZFNs), clustered regularly interspaced short palindromic repeats associated protein RNA guided nucleases (e.g. CRISPR-Cas9 system) and transcription-activator like effector nucleases (TALENs), are being used for site-specific transgene insertion (Malphettes et al., 2010; Le et al., 2015; Lee et al., 2015; Sakuma et al., 2015). These programmable endonucleases introduce DNA double strand breaks at a selected locus in the genome, and during the process of repairing this break, the cellular machinery may insert the transgene expression cassette at the break site by employing homologous recombination pathways. Therefore, both the exogenous endonuclease and the cellular repair mechanism are critical to the efficiency of this method (Turan et al., 2011; Kim and Kim, 2014).

Recombinase-Mediated Cassette Exchange (RMCE) using site-specific recombinase systems such as Cre-lox, Flp-FRT, Bxb1-attP/B and ΦC31-attP/B have also been used as genome engineering tools (Kito et al., 2002; Turan et al., 2013; 2014; Kawabe et al., 2015; Inniss et al., 2017; Chi et al., 2019). These enzymes can perform precise DNA recombination reactions at their respective cognate sites without a need for host factors and can lead to DNA segment insertions, deletions, or inversions (Akopian and Marshall Stark, 2005; Zhou and Droge, 2006). In RMCE, two different recombinases (e.g. Cre and Flp) are often employed to insert the transgene construct into an artificial genomic landing pad that carries the respective pair of recombination target sequences. The landing pad locus in the host cell chromatin should be accessible for both the recombinases and incoming transgenes. In addition, it must be genetically stable for sustained, high expression of transgenes. A number of these functional hotspots have been identified in CHO and in human cells (Hamaker and Lee, 2018; Aznauryan et al., 2022). Recently, another editing tool based on λ-phage integrase has been engineered for human genome manipulation especially for large transgene insertion reactions. The integrase was genetically modified by directed evolution to generate an enhanced, so-called IntC3 variant for mammalian cells (Siau et al., 2015), that works efficiently in the targeting of a novel endogenous human target sequence (Vijaya Chandra et al., 2015; Makhija et al., 2018; Chaudhari et al., 2020).

Most genome engineering approaches that aimed to increase production of biotherapeutics have been applied to CHO cells. The relatively new human Expi293F cell line, however, is mostly used for transient transgene expression. Since transient biotherapeutics expression is not always an attractive option from an industrial or cell-therapeutic perspective, an Expi293F platform should entail transgenic master cell lines with modular features as a basis for biopharmaceutical testing/production and innovative therapeutic applications such as transplantable cell-encapsulated mini bioreactors (Zhang et al., 2007; Lathuilière et al., 2015; Bose et al., 2020).

In this study, we presented such a versatile λ-integrase-based platform for site-specific transgenesis in Expi293F cells. A recombination-proficient genomic locus has been selected as a single copy artificial landing pad for λ-integrase-mediated transgenesis. Large transgenic vectors carrying heavy and light chain anti PD-1 monoclonal antibody transgenes in different orientations with respect to each other were inserted, thus permitting direct comparisons of PD-1 antibody expression yields from otherwise isogenic cells. The PD-1 protein is present on the surface of T cells and binds to the PD-1 ligand (PD-L1) expressed on cancer cells resulting in the inhibition of cancer cell killing by the immune cells. Monoclonal anti-PD-1 antibodies impede this interaction by binding to PD-1 as a promising novel anti-cancer strategy (Na et al., 2017; Tan et al., 2022). Our platform-generated PD-1 antibody-expressing cells were encapsulated to create cellulose-based mini bioreactors producing PD-1 antibodies for possible future allogeneic cell-based therapies.

## Materials and methods

### Expi293F cell culture

Expi293F cells were cultured in suspension Expi293 Expression Medium (Gibco, Life technologies) with 100 Units/ml of Penicillin and Streptomycin (Gibco, Life technologies) in 125 ml flasks in an orbital shaker incubator at 125 rpm and 37°C with ≥80% relative humidity and 8% CO_2_. A cell density of three million cells per ml was maintained and the seeding cell density was 0.3 million cells per ml. These cells were adapted to adherent culture following a previously reported protocol (Ecker et al., 2020). In brief, two million cells from the suspension culture were plated in Dulbecco’s Modified Eagle Medium (DMEM) supplemented with 10% foetal bovine serum (FBS), 1% l-glutamine and 100 Units/ml of Penicillin and Streptomycin (Gibco, Life Technologies) at 37°C under 5% CO_2_ in humidified conditions in a 10 cm tissue culture plate (TPP, Switzerland). For readaptation of cells from adherent to suspension culture, cells were detached using Trypsin-EDTA (Gibco, Life Technologies) from a confluent 150 cm^2^ tissue culture flask (TPP, Switzerland), and 15 million cells were suspended in 25 ml Expi293 Expression Media and placed in a 125 ml flask in an orbital shaker incubator. For selection of recombinants, hygromycin B (Invitrogen, Life Technologies) was used at 500 μg/ml. After selection, pure clones were expanded and transferred to flasks containing Expi293F expression media to further readapt them to suspension culture as previously described (Crescioli et al., 2018).

### Plasmids

Standard molecular cloning protocols were used to construct the plasmids used in this study. Q5^®^ High-Fidelity DNA Polymerase (NEB) was used for PCR amplifications and *E. coli* DH5α was used for plasmid preparation. The construction of the Int expression vectors (pCMVssInt-h/218 and -Int-C3) has been described previously (Vijaya Chandra et al., 2015). pEF_*attP*_mCherry was prepared by replacing the Neo_IRES_dTomato cassette with the mCherry_loxP cassette (PCR amplified using mCherry_BamHI and SV40_LoxPmCherry_HindIII primers listed in Supplementary Table S1) in pEF_*attP*_Neo_IRES_dTomato between the BamHI and HindIII sites.

The p*attB*_HygroR_eGFP plasmid was constructed by cloning the eGFP expression cassette into the NaeI site in p*attB*_HygroR by homologous recombination cloning using the ClonExpress Ultra One Step Cloning Kit (Vazyme, Nanjing, China) using the manufacturer’s protocol. The p*attB*_HygroR_PD1 HC and LC_PuroR_eGFP plasmids were constructed and obtained from e-Zyvec (Loos, France).

### Transfections

For suspension cultures, 5 × 10^5^ Expi293F cells were plated in 2 ml of Expi293 Expression Medium per well of a 6-well plate (TPP, Switzerland). Cells were transfected with 1ug of pEF_*attP*_mCherry using Lipofectamine 2000 (Invitrogen, Life technologies) using DNA: Lipofectamine ratios of 1 μg: 3 μL. For each transfection per well, complexes were prepared by mixing DNA and Lipofectamine reagent separately diluted in 100 μL of Opti-MEM medium (Life technologies) and incubating for 20 min at room temperature. The transfection mix was then added drop wise onto the cells and incubated overnight. The next day, the medium was changed after centrifugation at 400 g for 4 min. After 2 days, cells were suspended in 20 ml Expi293 Expression Media and transferred to 125 ml suspension culture flasks.

For transfection of adherent Expi293F cells, 3 × 10^5^ cells were seeded in 2 ml DMEM growth medium with 10% FBS per well of a 6-well plate (TPP, Switzerland) a day before transfection to obtain 70%–90% confluence at the time of transfection. Transfections for targeting in adherent cells were carried out with Lipofectamine 2000 or 3,000 (Invitrogen, Life technologies) using DNA: Lipofectamine ratios of 1 μg: 3 μL as described above, under antibiotics free DMEM growth medium with 10% FBS and transfection was allowed to proceed for 4–6 h before replacing with fresh growth medium.

### Antibiotic selection and screening for targeted cell clones in adherent culture

Forty-eight hours post transfection in adherent cells, selection with Hygromycin B in growth medium at 500 μg/ml (and for plasmids carrying the IgG genes also Puromycin at 1 μg/ml) was initiated. The selection medium was replaced every 2 days until colonies formed. At this stage, colonies were picked by carefully scraping patches of cells with a pipette tip and transferred to 24-well tissue culture plates for clonal expansion. The clones were sequentially expanded from 24-well to 6-well tissue culture plates. Genomic DNA was extracted using the DNeasy Blood & Tissue Kit (Qiagen, GmbH) as per the manufacturer’s protocol. Clones were further maintained without antibiotic selection in the culture media. Clonal cell lines were generated by serial cell dilution from the picked colonies.

### Identification of recombination events by PCR screening

PCR of DNA extracted from the clones (adherent or suspension cultures) was performed using GoTaq Flexi DNA polymerase (Promega) to amplify genomic recombination junctions using the primers listed in the figure descriptions and 500 ng of genomic DNA from each recombinant clone or parental cells as a template in 50 μL reactions. The thermal cycling parameters for the PCR was as follows: initial denaturation at 95°C for 2 min, 35 cycles of denaturation at 95°C for 1 min, annealing at 55°C for 1 min and extension at 72°C for 1 min per kb, and a final step of 72°C for 5 min. The PCR products were analysed by electrophoresis in 0.8% agarose (Seakem Agarose, Lonza, USA) gels in 0.5X TBE (Tris-Boric acid-EDTA buffer) containing 0.5 μg/ml ethidium bromide. PCR-generated products were compared with DNA standard markers and digitally documented under UV illumination (Quantum Vilber Lourmat, Germany). PCR-amplified products were analysed by sequencing. Primers are listed in Supplementary Table S1.

### Flow cytometry

A FACS Calibur Flow Cytometer (Becton Dickson) and CELL Quest software (Becton Dickson) were used to quantify mCherry+ and eGFP + cells. Cells were harvested and suspended in the corresponding media. A dot plot of side scatter (SSC) *versus* forward scatter (FSC) was used to gate live cells to separate them from aggregated and dead cells. For further analysis, mCherry *versus* FSC and mCherry *versus* eGFP plots were constructed for gated cells. Data was analysed using BD FACSDiva™ software, and mCherry^−^/mCherry^+^ and eGFP^−^/eGFP^+^ cells for each sample were indicated (as %) in each quadrant.

### Southern blot analysis

Southern blot probes were prepared using the PCR DIG Probe Synthesis Kit (Roche) as per manufacturer’s protocol. For the mCherry probe, mCherry_probe_fwd and 116_mCherry_rev primers were used, for the eGFP probe, 241_eGFP_probe_fwd and 242_eGFP_probe_rev primers were used and for the anti-PD1 IgG light chain probe, PD1LC_probe_fwd and PD1LC_probe_rev primers were used. Genomic DNA was purified using the DNeasy Blood & Tissue Kit (Qiagen, GmbH). 20 μg of genomic DNA was subjected to restriction digestion using 50 U of the respective enzyme in 200 μL overnight at 37°C. DNA was ethanol precipitated and dissolved in 20 μL TE buffer (pH 8.0). Target vectors were linearized with single cutter restriction enzymes and diluted to 10^7^, 10^8^ copies per μl. Digested genomic DNA samples were resolved overnight on a 0.8% agarose gel in 1X TAE (Tris-acetate–boric acid) buffer, with 1 kb DNA ladder (Thermo Scientific) and 1 μL of positive control samples. After transfer to the positively charged nylon membrane (Roche) by capillary transfer, Southern blotting employing the respective probes as indicated, was performed using the DIG-High Prime DNA Labelling and Detection Starter Kit II (Roche) as per the manufacturers’ protocol. The probe-target hybrids on the blots were detected by chemiluminescent assay using ChemiDoc MP (Biorad).

### Inverse PCR

Inverse PCR and subsequent nested PCRs were performed using GoTaq Flexi DNA polymerase (Promega). Genomic DNA was purified from isolated clones and 2 μg genomic DNA was digested with the restriction enzymes NheI, HindIII, PvuI, SgrAI, BsmI, BmtI, AgeI, EcoRI and MfeI overnight. The digested genomic DNA was purified by PCR purification kit (Qiagen). T4 DNA ligase (NEB) was used to self-ligate 250 ng of digested genomic DNA using 1 μL of enzyme in a 250 ul reaction volume to promote self-ligation with overnight incubation. Next day, ligated DNA was again purified using the PCR purification kit (Qiagen) and inverse PCR was performed with eluted DNA as follows: initial denaturation at 95°C for 2 min, 35 cycles of denaturation at 95°C for 1 min, annealing at 55°C for 1 min and extension at 72°C for 8 min, and a final step of 72°C for 10 min using the primers EF_rev_474 and 67_mCherry_fwd listed in Supplementary Table S1. Next, nested PCR was performed with 2 μL of inverse PCR product in a 50 μL reaction using same conditions with the EF_rev_104 and mCherry_fwd_597 primers listed in Supplementary Table S1. PCR products were resolved on 0.8% agarose gels and amplified bands were sequenced.

### IgG purification and SDS-PAGE

Cells from clones 6B1 and 23A4 were seeded at 0.5 million cells per ml density in 30 ml Expi293 Expression Medium in 125 ml culture flask and allowed to grow for five to 7 days until dead cells appeared. IgG secreted from clone 6B1 and 23A4 was isolated from the culture media after pelleting the cells at 500 X g for 5 min. The NAb™ Protein G Spin Kit (Thermo Scientific) was used following the manufacturer’s protocol. In brief, collected media was incubated with resin overnight in a cold room. Media was removed by centrifugation the next day and the resin was washed with the provided buffer followed by elution in the given solution. IgG was eluted in different fractions and the IgG concentration was measured using a NanoDrop™ 2000/2000c Spectrophotometer (Thermo Scientific). 15 μL from each eluted sample was prepared with 4X loading dye and resolved in Invitrogen Bolt 4%–12% Bis-Tris Plus precast gels (Thermo Fisher Scientific).

### Cell encapsulation

IgG secreting clone 6B1 cells were micro-encapsulated using the Cell-in-a-Box^®^ kit from Austrianova Singapore Pte. Ltd. following the manufacturer’s protocol. In short, about 0.8 million cells were suspended and mixed well into 1 ml of a proprietary sodium cellulose sulphate solution (Solution 1), drawn up into a syringe and a fine, blunt-ended needle added. The cell/SCS mixture was dropped into a constantly stirring gelation bath made up of a second polymer (Solution 2) at the rate of one to two drops per second. More than 30 capsules were obtained and incubated in Solution two for 5 min with constant stirring in order to create a stable membrane around the capsule. This step was followed by three PBS washes and three culture media washes. The capsules were then transferred by serological pipette into culture dishes and placed in an incubator with fresh media. The porous nature of the membrane allowed the capsules to be cultured over several days whilst the cells divided inside until the capsules were full with thousands of cells.

### IgG binding assays by ELISA

The amount of IgG secreted from encapsulated cells into media was estimated using a Human IgG ELISA kit (abcam) as per the manufacturer’s manual. In brief, 100 μL media from different samples of encapsulated cells was added into the wells of a 96-well plate provided in the kit and incubated for 2.5 h followed by several washing steps and incubation in biotinylated IgG solution for 1 h. After further washing, plates were incubated with HRP-streptavidin solution for 45 min. This was followed by further washing and incubation with TMB substrate reagent for 30 min. A microplate reader was used to quantify emissions at 450 nm after adding stop solution. All incubations were performed at room temperature with constant shaking.

Biotinylated recombinant human PD-1 (Sino Biological# 10377-H08H) was immobilized on the neutravidin (ThermoFisher scientific, #31000)-coated ELISA plate, to which the anti-PD-1 antibodies at the indicated concentrations were added. Following incubation and washes, peroxidase-conjugated F (ab')2 Fragment Goat Anti-Human IgG (JACKSON ImmunoResearch, #109-036-098) at 1:3,000 dilution was added to each well and the samples were incubated at room temperature for an hour. The wells were then washed and the substrate TMB (SurModics, #TMBW-1000-01) was added. The reactions were stopped by adding half volume of 1M HCl and the absorbance at 450 nm was measured on a microplate reader.

## Results

### Generation of Expi293F cell lines with single-copy artificial docking sites

**FIGURE 1:**
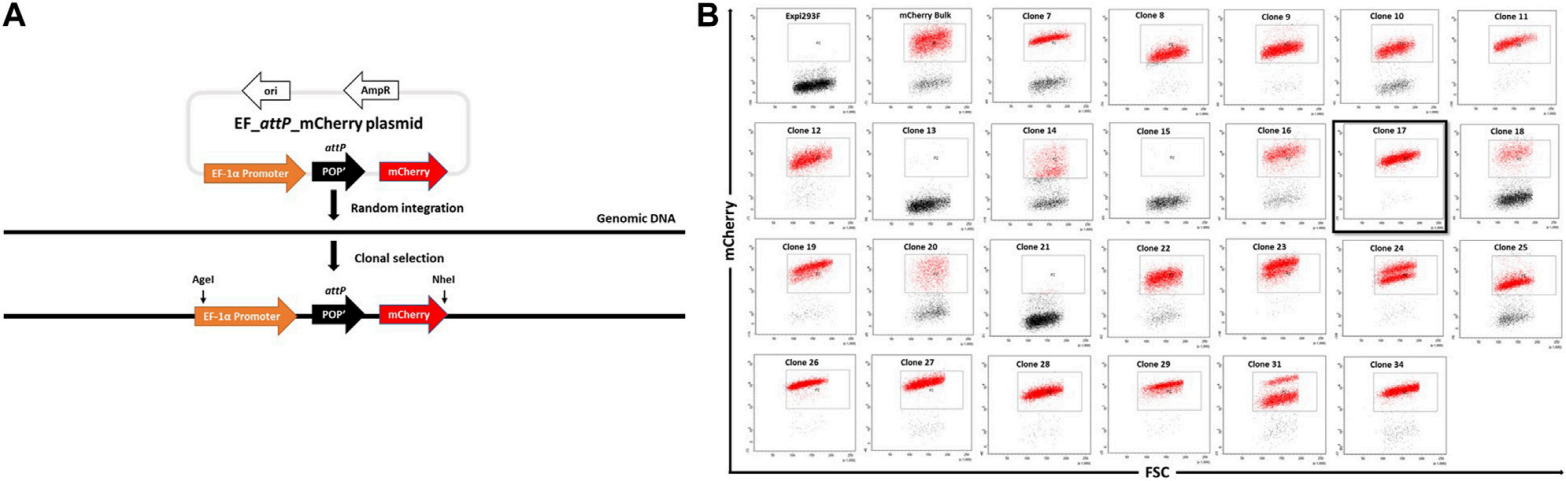
Generation of landing-pad inserted clones. **(A)**. An illustration of pEF_*attP*_mCherry and of the experimental strategy for creating EF_*attP*_mCherry or landing pad inserted clonal Expi293F cells. **(B)**. Screening of mCherry positive clones. Expi293F cells were sorted for mCherry positive fluorescence after transfection with pEF_*attP*_mCherry. Once a stable population had been obtained, single cell clones were obtained by dilution. Single cell clones were next analyzed by flow cytometry. Clone 17 (black border) was selected for further experiments.

We devised a strategy to generate clonal Expi293F cell lines containing a single copy landing pad for λ-integrase-mediated insertion of large transgene constructs (Figure 1A). The eukaryotic elongation factor 1 alpha promoter (pEF-1α) and the coding region for fluorescent protein mCherry flank the recombination target sequence *attP* (241 bp) in plasmid pEF_attP_mCherry. The *attP* site lacks translational start codons thus enabling mCherry expression from the upstream pEF-1α promoter.

Expi293F cells in suspension culture were transfected with pEF_*attP*_mCherry and maintained with regular passages in the absence of selection pressure. mCherry^+^ cells were enriched by several rounds of bulk fluorescent cell sorting. Hence, after random integration, a stable mCherry^+^ expressing bulk cell population was obtained. To generate monoclonal mCherry^+^ cell lines, cells were adapted to adherent growth (Crescioli et al., 2018) as adherent cell culture offers advantages in downstream processes like colony picking and expansion of single cells in 96-well plates.

Adherent cells from the mCherry^+^ bulk population were serially diluted to attain single cells which were expanded with the aim to obtain monoclonal cell lines with stable and uniform mCherry expression as analysed by flow cytometry (Figure 1B). Several clones showed homogenous mCherry^+^ expression, while a few, such as clone #24, showed two populations of cells with distinct mCherry expression levels most likely indicating the presence of two different population of cells each with a different transgene copy number. Based on this analysis, we selected clone #17 due to its high homogeneity and narrow mCherry expression pattern which presumably came from a single copy transgene.

### Targeted integration into attP of Expi293F cells

**FIGURE 2:**
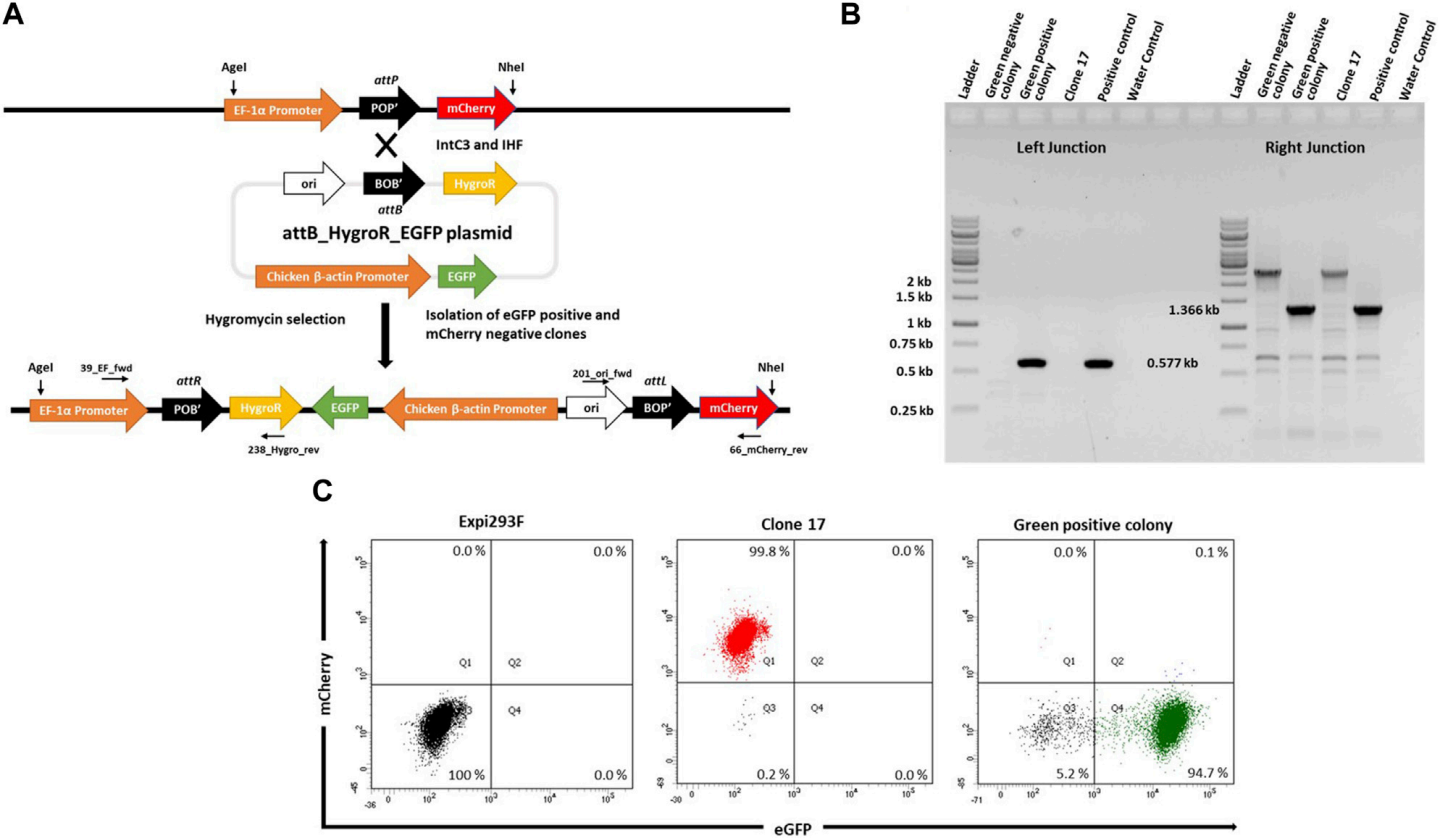
Targeted integration of p*attB*_HygroR_eGFP landing pad. **(A)**. Schematic figure showing intC3 facilitated *attP* X *attB* recombination and targeted integration of p*attB*_HygroR_eGFP at the landing pad and predicted recombination construct and positions of PCR primers: 39_EF_fwd and 238_Hygro_rev (Left junction) and 201_ori_fwd and 66_mCherry_rev (Right junction). **(B)**. PCR confirmation of *attP* X *attB* recombination resulting in *attR* and *attL* sites at the left (0.577 kb) and right (1.366 kb) junctions, respectively. PCR was performed with genomic DNA as a template from a green negative, a green positive colony and clone 17 with the mentioned primers. Genomic DNA from bulk targeted and antibiotic selected cells was used as positive control and no template DNA as water control. Ladder denotes 1 kb DNA ladder. **(C)**. Flow cytometric analysis of the selected colony. Dot plots representing mCherry negative and eGFP negative Expi293F cells in the lower left quadrant in the first panel, mCherry positive and eGFP negative clone 17 cells in the upper left quadrant in the second panel and mCherry negative and eGFP positive cells from green positive colony in the lower right quadrant in the third panel.

In order to target the *attP* site in mCherry^+^ cells, we generated a target vector containing an *attB* site as the corresponding recombination partner sequence (21bp) for genomic attP and a downstream promoter-less hygromycin resistance gene plus an enhanced green fluorescent protein (eGFP) expression cassette driven by the Chicken *ß*-actin promoter (Figure 2A; pattB_HygroR-EGFP plasmid). This target vector was co-transfected with expression plasmids for λ-Integrase variant IntC3 and the single chain integration host factor 2 (scIHF2; as an optional IntC3 recombinase co-factor; (Corona et al., 2003)). Successful integration events catalysed by the recombinase should result in the insertion of the target vector leading to expression of hygromycin^r^ and eGFP^+^, the cessation of mCherry expression, and the creation of two genomic recombination junction sequences attR and attL, as depicted in Figure 2A.

To demonstrate functionality of the attP site as a transgene landing pad in clone #17, co-transfections were performed followed by hygromycin selection. We expected that surviving colonies would be either double positive for mCherry and eGFP, or single positive for either eGFP or mCherry. The mCherry-expressing cells would most likely entail untargeted cells that carried truncated target vectors resulting in hygromycin resistance. The double positive cells would be either untargeted cells with non-specifically integrated target vectors or successfully targeted cells if there were more than a single landing pad construct present in the genome of parental clone #17 cells.

To confirm successful targeting, individual eGFP^+^ and eGFP^−^ colonies were expanded for junction PCR analysis as depicted in Figures 2A,B. Genomic DNA from these colonies was subjected to PCR analysis using the primers 39_EF_fwd and 238_Hygro_rev for the attR junction, and the primers 201_ori_fwd and 66_mCherry_rev for the attL junction. The presence of a 0.577 kb product (Figure 2B, Green positive colony; Left Junction) and of a single product of 1.366 kb (Figure 2B, Green positive colony; Right Junction) confirmed that singly eGFP^+^ cells had been successfully targeted (Figure 2B). Faithful recombination by IntC3 was confirmed by sequencing of the PCR products (Supplementary Figure S1). Flow cytometry of these eGFP^+^ cells revealed a highly homogenous (>90%) eGFP^+^ cell population and the absence of mCherry^+^ cells (Figure 2C). We conclude that upon successful targeting of the attP landing pad in clone #17 cells, eGFP expression completely replaced mCherry expression. This feature can be utilized for the selection of stably targeted cells for future applications.

To evaluate the targeting efficiency of attP in #17 cells using IntC3, we next transfected the same target vector with and without IntC3 expression plasmid. After hygromycin selection, cells were analysed as bulk by flow cytometry to determine the fraction of eGFP^+^/mCherry^−^ cells. The results showed that only a negligible number of cells were found to be eGFP^+^ after transfection with target vector alone (Supplementary Figure S2, no integrase; Q4), while >40% eGFP^+^ cells were obtained after co-transfection with the IntC3 plasmid (Supplementary Figure S2, IntC3; Q4).

### Expi293 clone #17 cells harbour a single functional landing pad site on chromosome two

**FIGURE 3:**
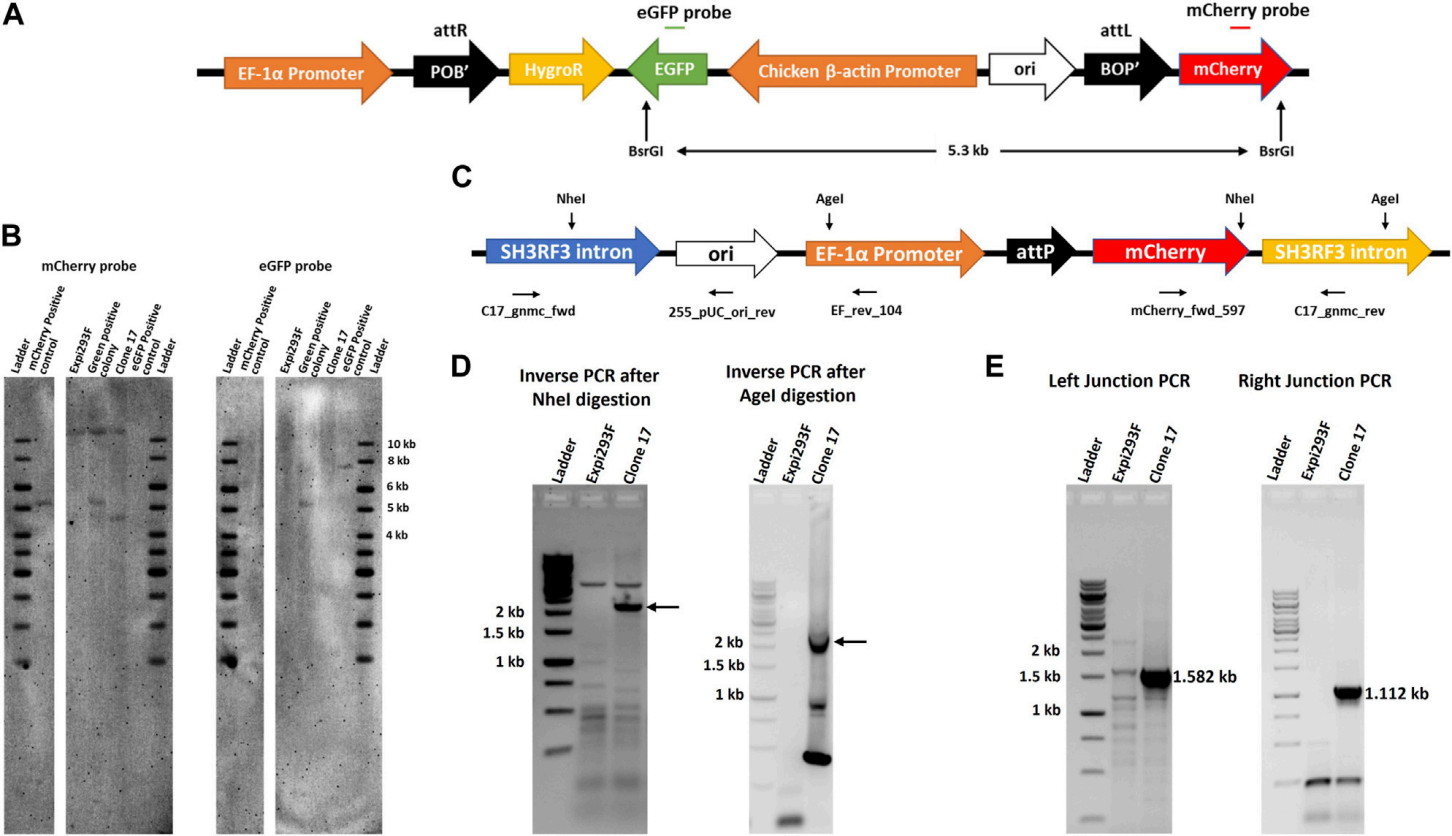
Single copy of landing pad in chromosome two of clone 17. **(A)**. Schematic representation of p*attB*_HygroR_eGFP integrated construct with positions of BsrGI restriction sites and of the ∼5.3 kb predicted product after digestion. **(B)**. Southern blot confirmation of single landing pad site. Southern blot was performed with BsrGI digested genomic DNA from Expi293F, clone 17 and green positive colony cells and incubated with a mCherry gene probe followed by an eGFP gene probe after stripping the same blot. As a positive control 0.5 million copies of pEF_*attP*_mcherry (5.217 kb) and p*attB*_HygroR_eGFP (7.550 kb) were used after linearization by BsrGI. **(C)**. Schematic drawing of the landing pad site in the SH3RF3 intron of chromosome two of clone 17 depicting NheI and AgeI restriction sites with the primers used for nested PCR after inverse PCR and junction PCR. **(D)**. Detection of the specific site of landing pad insertion by inverse PCR. Inverse PCR and nested PCR were performed, after NheI (for left junction or EF promoter side) or AgeI (for right junction or mCherry side) digestion of clone 17 genomic DNA, with EF_rev_104 and mCherry_fwd_597 primers and nested PCR products were resolved on an agarose gel. DNA bands marked with an arrow were excised and extracted DNA was sequenced to identify the site of the landing pad insertion in the genome. **(E)**. Genomic location confirmation by junction PCR. Junction PCR was performed with clone 17 genomic DNA using C17_gnmc_fwd and 255_pUC_ori_rev primers for the left junction or mCherry_fwd_597 and C17_gnmc_rev primers for the right junction. Amplified products were sequenced to confirm the site of insertion. Ladder denotes 1 kb DNA ladder.

Our results indicated that the artificial attP landing pad can be efficiently targeted by IntC3 in clone #17. We next performed Southern blot analysis to determine the copy number of attP sites. Genomic DNA from targeted (eGFP^+^) and untargeted (mCherry^+^) clonal cells was digested with the enzyme BsrGI, which is expected to cut two times in the transgenic DNA after integration of the target vector into the landing pad to generate a 5.3 kb fragment containing the eGFP, Chicken ß-actin Promoter, ori, BOP’ and mCherry sequences (Figure 3A). We employed mCherry and eGFP probes to determine the copy number of landing pads in parental clone #17, as well as the number of transgenes after targeting the landing pad in clone #17 (Green positive colony). As expected, a signal at 5.3 kb genomic fragment size was obtained with both probes in eGFP^+^ cells indicating that a single targeted attP-mCherry cassette is present in the genome (Figures 3A,B). This conclusion was corroborated by the detection of a fragment of about 4.5 kb (Figure 3B, mCherry probe, clone #17) using the mCherry probe in the untargeted cells. This fragment resulted from restriction cleavage at the end of the mCherry sequence and at an unknown locus within the genomic DNA. As expected, no fragment was detected in parental untargeted cells using the eGFP probe (Figure 3B, eGFP probe, clone #17).

Having demonstrated that parental #17 cells contained one copy of the randomly inserted pEF_*attP*_mCherry cassette, inverse PCR was employed to identify the exact genomic location of the landing pad cassette. Genomic DNA was digested with either the restriction enzyme NheI or AgeI which have single sites within the landing pad sequence (Figure 3C), purified and self-ligated, followed by inverse PCR using EF_rev_104 and mCherry_fwd_597 primers. NheI digestion and self-ligation followed by inverse PCR yielded a >2 kb product (Figure 3D, Inverse PCR after NheI digestion, clone #17) which should contain flanking genomic sequences located 5′ of the EF-1α promoter. AgeI digestion and self-ligation followed by inverse PCR yielded a ∼2 kb long product (Figure 3D, Inverse PCR after AgeI digestion, clone #17) which should contain flanking genomic sequences located 3’ of the integrated mCherry coding sequence. Sequencing of these PCR products revealed the same genomic locus as integration site of the landing pad cassette. Inverse PCR analyses after genomic DNA digestion with other restriction enzymes further corroborated these results (Supplementary Figure S3). Subsequent nucleotide sequence alignments revealed that the genomic locus of the landing pad cassette was identified in the third intron of the SH3 Domain Containing Ring Finger three gene (*SH3RH3*) on chromosome 2 (Figure 3C, SH3RF3 intron). The sequence alignment also revealed that the random pEF_*attP*_mCherry cassette integration resulted from a DNA double strand break in that intron with loss of only six nucleotides as depicted in Supplementary Figure S3.

To directly confirm the sequence accuracy of the targeted genomic locus for landing pad insertion, direct genomic junction PCRs were performed on genomic DNA using primers C17_gnmc_fwd located in the *SH3RH3* intron along with primer 255_pUC_ori_rev to obtain the predicted size for the left junction PCR product of 1.582 kb (Figure 3E; Left Junction PCR, clone #17), and by using primers C17_gnmc_rev located in the *SH3RH3* intron along with primer mCherry_fwd_597 to obtain the predicted size for the right junction PCR product of 1.112 kb (Figure 3E; Right Junction PCR, clone #17). Sequencing results confirmed the site of insertion and break points in the plasmid (Supplementary Figure S4).

### Targeted expression of monoclonal anti PD-1 antibodies in Expi293F cells

**FIGURE 4:**
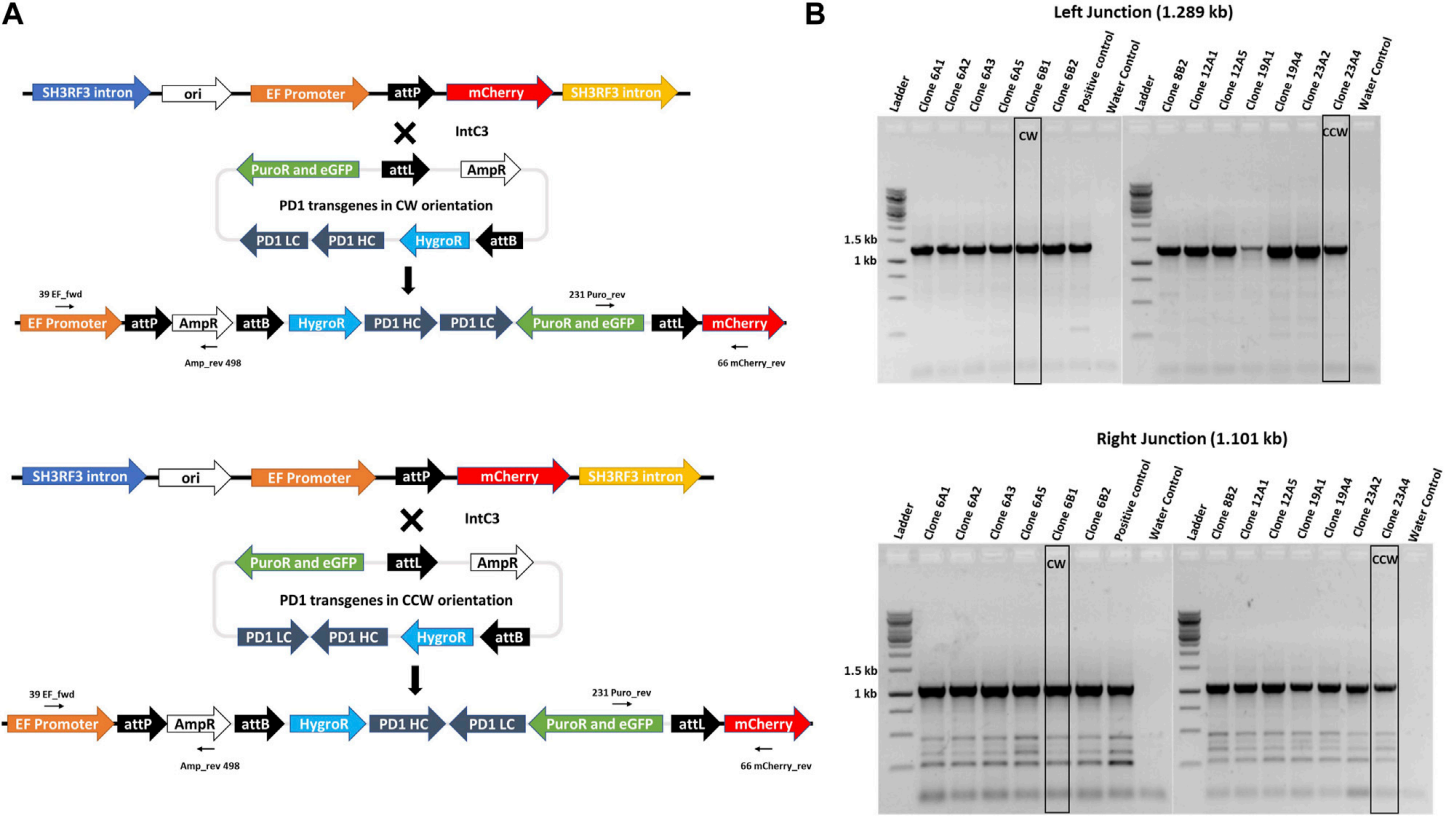
Targeted integration of IgG genes containing plasmids at the landing pad in clone 17. **(A)**n il. Alustration of *attP* X *attL* recombination between the landing pad and anti-PD1 IgG heavy and light chain genes containing plasmid with either CW or CCW orientations. Predicted integrated constructs are depicted with the primers used to confirm integration by junction PCR analysis. **(B)**. PCR confirmation of the left and right junctions. PCR was performed with genomic DNA from different subclones of clones 6, 8, 12, 19 and 23 using either 39_EF_fwd and Amp_rev_498 primers for the left junction with an expected 1.289 kb product or 231_Puro_rev and 66_mCherry_rev primers for the right junction with an expected 1.101 kb product. Bands obtained after resolution on an agarose gel were later confirmed by sequencing. Clones 6B1 and 23A4, marked by black border, were further used for protein expression. Ladder denotes 1 kb DNA ladder.

To exemplify utility of our Expi293F transgenic cell platform, we generated complex multi-transgene target vectors harbouring anti-PD-1 human IgG heavy and light chain monoclonal antibody genes (Wang et al., 2015) expressed from different promoters. Heavy (PD-1 HC) and light (PD-1 LC) chains were placed in two orientations with respect to each other, i.e. head-to-tail (CW) or head-to-head (CCW) (Figure 4A), to evaluate a possible impact on antibody expression. In addition, these targeting plasmids also contain a puromycin resistance gene for stringent selection, an eGFP gene as a marker and an *attL* recombination site. The promoter-lacking hygro^r^ gene is located downstream of a second recombination site, attB. These plasmids were designed to simultaneously test which recombination site pairing (i.e. genomic attP x vector attB; vector attL x vector attB; genomic attP x vector attB) would ultimately yield targeted genome insertion in parental clone #17 cells. IntC3-mediated intramolecular recombination between *attL* and *attB* sites that occur before intermolecular genomic insertion would result in seamless vectors as previously described (Makhija et al., 2018).

Adherent #17 cells were co-transfected with PD-1 target vectors (antibody genes either in CW or CCW orientation) and IntC3 plus scIHF2 expressing plasmids. After antibiotic selection, 26 colonies that initially appeared eGFP^+^ and mCherry^−^ were isolated, expanded and screened for attP-targeted genomic integration using specific PCR primer combinations that considered the different possibilities of att site pair utilization as mentioned above. We confirmed targeted attP site transgene insertion on chromosome two in eight colonies, with four colonies carrying the PD-1 genes in CW orientation and four colonies in CCW orientation (Supplementary Figure S5). Surprisingly, we found that all eight carried the transgene cassette as a result of IntC3-mediated *attP* (genome) X *attL* (vector) recombination events (Figure 4A). This result thus revealed a very strong preference for this pair of att sites for targeted genome integration in attP since parallel PCR screening of the 26 colonies for attP (genome) x attB (vector) recombination events produced no positive results.

Antibiotic selection was removed from targeted colonies, and pure cell sub-clones from both target vectors were obtained by two-fold serial cell dilution from clones 6 (CW) and 23 (CCW) (Supplementary Figure S5). Faithful transgene insertion resulting from attP (genome) x attL (vector) site-specific recombination was confirmed in all 13 sub-clones analysed by PCR/sequencing of both junctions (Figure 4B, Supplementary Figure S6). One individual sub-clone obtained with each target vector, i.e. #6B1 (CW) and #23A4 (CCW), was selected for detailed PD-1 antibody expression analysis.

**FIGURE 5:**
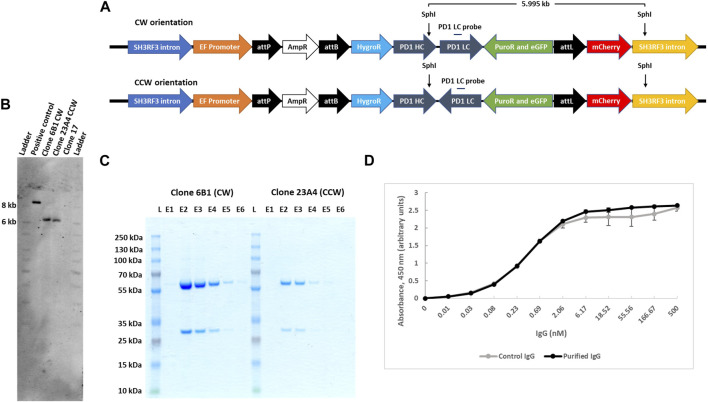
IgG expression and purification from landing pad targeted clones. **(A)**. Schematic presentation of IgG genes carrying plasmids, with either CW or CCW arrangement, integrated at the landing pad in clone 17. SphI restriction sites are shown in the figure that would yield a 5.995 kb product after digestion of the genomic DNA clones with both CW or CCW arrangement of the transgenes. **(B)**. Southern blot confirmation of the single copy integration of IgG transgenes for both orientations. Southern blot was performed with SphI digested genomic DNA from clone 17, 6B1 (CW) and 23A4 (CCW) cells and analyzed with an IgG light chain gene probe. As a positive control, 0.5 million copies of targeting plasmid with CW arrangement (10.793 kb) were used after digestion with SphI, yielding an 8.468 kb fragment when hydridized to the light chain gene probe. **(C)**. Purification of secreted IgG. Secreted IgG was purified from both clone 6B1 and 23A4 media by protein G agarose resin and an equal volume (30 μL) from each eluant was resolved on SDS-PAGE. **(D)**. PD-1 antibody binding assay. The functionality of the combined monoclonal IgG fractions was analysed by ELISA using immobilized human PD-1 protein. Purified monoclonal anti-PD1 IgG produced by transiently transfected CHO cells was used as control. Data presented are mean of two technical repeats.

IntC3 has previously been shown to site-specifically insert large (>8 kb) plasmids into the human genome (Vijaya Chandra et al., 2015). Here, we have been able to demonstrate that a >10 kb plasmid containing five transgenes could be successfully delivered. To confirm the accuracy of the inserted PD-1 transgene constructs, PCR and sequencing analysis was used on the two selected sub-clones #6B1 and #23A4 and revealed that both clones had the correct internal sequence indicative of their transgene orientations (CW or CCW) without cross-contamination (Supplementary Figure S7A). Both cell clones were further subjected to Southern blot analysis to verify single copy plasmid integration at the genomic attP target site on chromosome two using a IgG light chain gene probe. Genomic DNA was digested with SphI which yields a single diagnostic fragment of 5.995 kb for both PD-1 gene orientations (Figure 5A). This fragment was detected in both analysed subclones (Figure 5B, clone 6B1 and clone 23A4) but not in the parental cell line containing the untargeted landing pad (Figure 5B, clone 17). The appearance of a single fragment thus confirmed single copy integration since the downstream SphI site lies within the predicted genomic (*SH3RF3* intron) sequence flanking the landing pad.

We next compared levels of antibody secretion for the two selected subclones which differed in heavy and light chain gene arrangements but were otherwise isogenic. Secreted IgG was affinity purified from the cell culture medium *via* stepwise elution from protein G agarose resins. The purity of eluted fractions was analysed by SDS-PAGE (Figure 5C; E lanes), and the total combined IgG yield determined by spectrophotometry. Isolated IgG from both clones was found to be homogeneous without visible contaminations. We found that from a small suspension culture volume (22 ml), IgG yield from clone #6B1 was about 4-fold higher than from clone #23A4 (41.75 ± 0.25 μg *versus* 10.35 ± 1.25 μg). Both cell sub-clones were also analysed by flow cytometry and were found to be more than 98% single eGFP^+^ (Supplementary Figure S7B).

In order to demonstrate that the secreted antibodies were functional in binding PD-1 antigen, we performed ELISA using immobilized biotinylated recombinant human PD-1. As control, we included the same PD-1 monoclonal antibody purified from CHO cells after transient transfection of expression vectors. The results clearly revealed that PD-1 monoclonal antibodies produced and secreted from our engineered transgenic clone #17 Expi293 cells are functionally indistinguishable from those synthesized from CHO cells (Figure 5D).

### Long-term homogeneous and selection-free transgene expression

For several downstream applications, it is critical that cells harbouring transgenes remain stable over long periods in the absence of any selection pressures. To determine genetic and epigenetic stability, cells from a p*attB*_HygroR_eGFP targeted colony were examined by flow cytometry before and after 14 days of continuous culture and were found to maintain homogeneity at > 90% (Supplementary Figure S8A). Similarly, FACS analysis showed sustained and homogenous (>98% eGFP^+^) in PD-1 antibody expressing sub-clones #6B1 and #23A4 after 14 days of culture without selection (Supplementary Figure S8B), indicating that integration at this landing pad locus on chromosome two is genetically stable as well as transcriptionally active over a long period of time. In fact, since the generation of these PD-1 expressing subclones was already performed in the absence of any selection pressure over more than 6 weeks, the transgene expression from this genomic locus remains unchanged over several months.

### IgG secretion from encapsulated Expi293F platform cells

Our Expi293F #17 cell platform can be used to engineer cells for perennial expression and secretion of biologics. Although the transgene`s genomic locus is well defined and genetically and functionally stable, expression levels from a single transgene will generally be lower than transient expression from multi copy episomal plasmids. However, finely tuned and steady expression could become important for other applications, such as cell therapy using mini-bioreactors comprising encapsulated cells engineered for therapeutic secretion (Pelegrin et al., 2000; Lathuilière et al., 2014; Lathuilière and Schneider, 2016; Hsu et al., 2022). These mini-bioreactors can secrete and accumulate biotherapeutics at high concentrations at the site of transplantation in the body. Encapsulation of cells prevents their escape from the site of implantation and it also protects them from the patient’s immune responses. The capsules also act as a safety device as they protect the patient from the foreign cells implanted.

**FIGURE 6:**
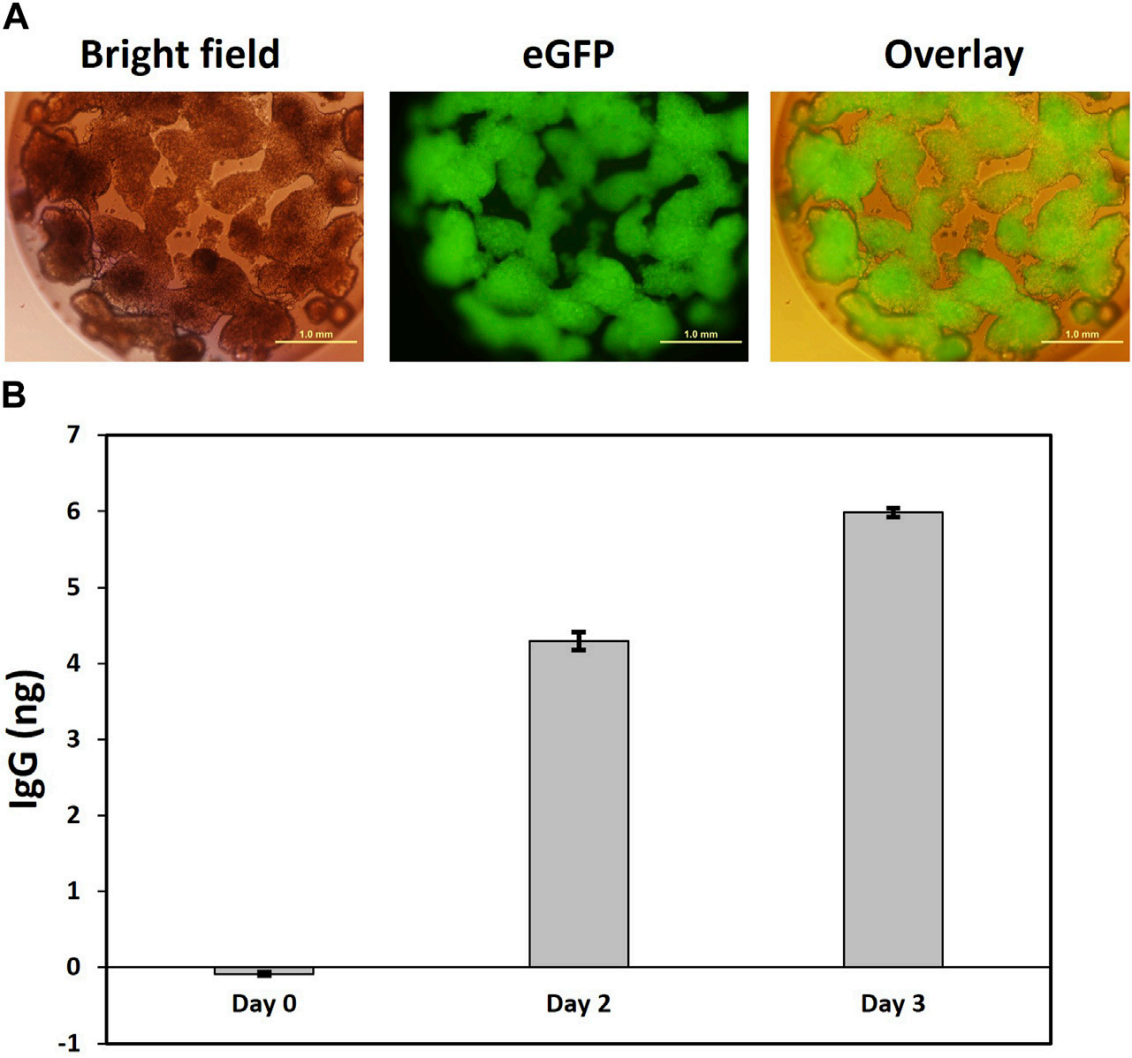
Secretion of IgG from encapsulated clone 6B1 cells. **(A)**. Representative image of capsule four in bright field and fluorescence showing homogenous eGFP expression in encapsulated cells. Encapsulation of clone 6B1 cells. Cells were encapsulated in Cell-in-a-Box^®^ and transferred to a 24-well plate with one capsule per well. This was followed by visualization under a fluorescence microscope 2 weeks after encapsulation. Scale bars represent 1 mm at ×4 magnification. **(B)**. Measurement of secreted IgG from capsule 4.20 days after encapsulation, the media in the well with capsule four was replaced with fresh media, which was defined as Day 0. Samples were then collected on Day 0, Day 2 and Day 3. The concentration of secreted IgG was estimated by ELISA in triplicate. Data presented here is the mean with standard deviation.

We employed here a well-established cellulose sulfate-based encapsulation strategy (Cell-in-a-Box^®^) that permits long-term monoclonal antibody secretion from encapsulated producer cells (Pelegrin et al., 2000; Löhr et al., 2001; Salmons et al., 2007). This proven method was used to encapsulate anti-PD1 antibody producing #6B1 cells, and we examined individual capsules for eGFP expression. As shown in Figure 6A with one isolated capsule as an example, eGFP + cells, which had been encapsulated at low cell density of about 5 k cells/capsule, continued to proliferate and eventually occupied previously empty spaces within capsules. Secretion of human anti PD-1 IgG from the encapsulated cells into the media was quantified by ELISA and revealed a steady 50% increase of IgG secretion per day (Figure 6B).

## Discussion

The rapid emergence of novel biologics warrants more time- and cost-effective cell-based tools for production. In this context, GMP-grade Expi293F cells of human origin are an increasingly attractive alternative to the currently used CHO cell lines. The development of Expi293F cell lines combined with safe genome editing tools to produce master clones provides additional advantages and has been the aim of this study.

Targeted genome manipulation by site-specific recombinases such as IntC3 employed here can enable precise, locus-specific knock-in of transgene expression constructs into stable and well-characterized sites in the host cell genome. In this study, we have devised a strategy to first select cell clones based on homogenous and stable fluorescent reporter expression in the absences of any selection pressure. This provided a simple screening method to subsequently derive master cell lines carrying a functional single copy artificial transgene landing pad, attP, by switching off fluorescent marker expression and establishing conditional antibiotic resistance after recombinase-mediated transgene insertion.

Here we presented the fully characterized master Expi293F cell line #17 which carries attP in an intron of the *SH3RF3* gene on chromosome 2 as a proof of concept. The SH3 domain containing ring finger three protein, SH3RF3, has E3 ubiquitin-protein ligase activity and is involved in JNK signalling and protein autoubiquitination (Zhang et al., 2020; Fu et al., 2021). The corresponding mRNA is found to be enriched in a few cell types such as in the kidney (Uhlén et al., 2015). There appears to be no compelling evidence that aberrant expression of this protein is causally linked to human pathologies, which renders the unique attP site in these cells as a potential safe harbour for transgene insertion. Furthermore, our characterization of targeted cells clearly revealed long-term genetic transgene stability and expression from the targeted genomic locus which only lacks six endogenous nucleotides in the *SH3RF3* gene. The availability of GMP-grade Expi293F cells in combination with programmable endonuclease-induced homologous recombination should enable the future generation of a GMP-based #17 master cell line homologue for clinical applications.

As an example of the utility of our #17 Expi293F cell line, we inserted two distinct large (>10 kb) transgene vectors for monoclonal anti PD-1 antibody expression into the genomic landing pad attP. We confirmed that resulting cell subclones carried only one intact copy of the respective transgene construct which differed solely in the orientation of heavy and light chain transcription units with respect to each other. Our quantitative antibody expression/secretion analysis revealed that a 4-fold higher antibody production is achieved when the two transcription units are oriented as direct (head-to-tail) repeats as opposed to the inverted (head-to-head) orientation at the same genomic locus. A possible explanation for such a pronounced difference could be transcription-induced DNA supercoiling which would lead to high levels of positive DNA supercoil accumulating in front of the two translocating RNA polymerases moving towards each other in the head-to-head gene orientation. This, in turn, may lead to inhibition of mRNA synthesis (Dröge, 1994).

Furthermore, our targeting of the single copy attP site with our antibody expression constructs revealed that the preferred recombination partner sequence for the genomic attP on the incoming target vector is attL. We demonstrated this by placing both attB, which is the natural recombination partner for attP in the wild-type phage lambda recombination system, and attL on the target vector thus creating competition for recombination with attP on chromosome 2. While we clearly demonstrated that attB can be used efficiently by IntC3 to recombine with attP in #17 cells when there is no attL site present (Supplementary Figure S2), it appears that attL under competing conditions is the preferred attP partner. Likewise, attL appears to preferentially recombine *in trans* with genomic attP instead of attB, even when attB is present *in cis* on the same DNA molecule. The reasons for these surprising *in situ* att site preferences of the IntC3 recombinase are currently not known.

For future therapeutic purposes, we encapsulated anti PD-1 antibody expressing cells to create cellulose-based mini-bioreactors and confirmed continued, steady IgG secretion from the capsules. Future applications could utilize allogeneic transplantation of such bioreactors into a patient`s body to deliver, for example, high antibody concentrations near sites of malignant cell growth. Considering the high costs of immunotherapies currently in clinical practice, mini-bioreactors could provide a safe and cost-effective alternative and have recently been shown to work efficiently in animal models (Bose et al., 2020; Nash et al., 2022a). In this context, our core platform offers future inclusion of additional site-specific recombinases as part of a modular system. For example, transgenic cell lines lacking remaining marker sequences for bioreactor production could easily be obtained by co-placing directly repeated pairs of each recombinase cognate sites loxP and FRT in the transgenic locus on chromosome 2. The Cre and Flp recombinases expressed from transfected mRNA can then excise undesirable DNA segments as modelled in Supplementary Figure S9. In addition, a single cognate sequence for the serine recombinase Bxb1, which works efficiently in human cells (Xu et al., 2013), can be included in the design. After IntC3-mediated recombination into the genomic attP, Bxb1-mediated intermolecular recombination can deliver an additional transgene construct into the same genomic locus to achieve, for example, tightly regulated large scale expression of (multi-)transgenes that exhibit severe mammalian cell toxicity.

## Data Availability

The original contributions presented in the study are included in the article/Supplementary Material, further inquiries can be directed to the corresponding authors.

## Abbreviations

CHO, Chinese hamster ovary (CHO) cells; EF or EF-1α, elongation factor-1 alpha; HEK, human embryonic kidney cells; mCherry, monomeric Cherry; eGFP, enhanced green fluorescent protein; HygroR, hygromycin resistance gene; PuroR, puromycin resistance gene; IntC3, λ-integrase C3; IgG, immunoglobin G; PD1, Programmed cell death protein 1.
