# Supplementary material for: A versatile genomic transgenesis platform with enhanced λ integrase for human Expi293F cells
Source: Front Bioeng Biotechnol. 2023 Jun 22;11:1198465. doi: 10.3389/fbioe.2023.1198465 (PMC10325659; doi:10.3389/fbioe.2023.1198465)
Supplement: Supplementary file 1 [file DataSheet1.pdf]

Supplementary Material

A versatile genomic transgenesis platform with enhanced λ integrase for human Expi293F cells

Asim Azhar Siddiqui[1], Sabrina Peter[1], Eve Zi Xian Ngoh[2], Cheng-I Wang[2], Shirelle Ng[3], John A. Dangerfield[3], Walter H. Gunzburg[3,4], Peter Dröge[1]* and Harshyaa Makhija[5]**

[1]School of Biological Sciences, Nanyang Technological University, Singapore 637551, Singapore

[2]Singapore Immunology Network, Agency for Science, Technology and Research (A*STAR), 8A Biomedical Grove, Immunos, Singapore 138648, Singapore

[3]Austrianova Singapore Pte Ltd, 41 Science Park Road, The Gemini #03-15, Singapore 117610, Singapore

[4]University of Veterinary Medicine, Vienna, Institute of Virology, Dept of Pathobiology, Veterinaerplatz 1, A-1210 Vienna, Austria

[5]LambdaGen Pte Ltd, No. 2, Havelock Road, #04-20 Havelock 2, Singapore 089066, Singapore

*** Correspondence:**
****** Corresponding Author at:
harshyaa@lambdagentherapeutics.com

Supplementary Table 1: Primers

	Primers	Sequence
1	39_EF_fwd	GTCCAGGCACCTCGATTAGTTCTC
2	238_Hygro_rev	CGAAGCTGAAAGCACGAGATTCTTC
3	201_ori_fwd	GATAAGTCGTGTCTTACCGGGTTG
4	66_mCherry_ rev	CTTGTAGATGAACTCGCCGTCCTG
5	mCherry_probe_ fwd	ATGGTGAGCAAGGGCGAGGAGG
6	116_mCherry_ rev	GCTTCAAGTAGTCGGGGATGTCG
7	241_eGFP_ probe_fwd	CCGACCACATGAAGCAGCACG
8	242_eGFP_ probe_rev	GTTCTGCTGGTAGTGGTCGGCG
9	EF_rev_474	AATGGCTAGAGACTTATCGAAAGCAGC
10	69_mCherry_ fwd	CGACATCCCCGACTACTTGAAGC
11	67_mCherry_ fwd	CGACATCCCCGACTACTTGAAGC
12	EF_rev_104	TACACGACATCACTTTCCCAGTTTACCC
13	mCherry_fwd_5 97	GTCAACATCAAGTTGGACATCACCTCC
14	C17_gnmc_fwd	GTAGTTTGGGTTTGTTTGTCTCCAGC
15	255_pUC_ori_ rev	CTTTCTCATAGCTCACGCTGTAGG
16	C17_gnmc_rev	GAAGTGAAAGGGAAGACAAGAACATGG
17	Amp_rev_498	CGTCGTTTGGTATGGCTTCATTCAG
18	235_Puro_rev	GAGGAAGAGTTCTTGCAGCTCGGTG
19	231_Puro_rev	GGAACCGCTCAACTCGGCCATGC
20	PD1LC_fwd	TGACGGTACTGGGACAACCTAAAGC
21	PD1HC_fwd	GACTACGGGGCAGGTCCTTACTACT
22	PD1LC_probe_ fwd	CCAAAGCGTCCTCACTCAACC
23	PD1LC_probe_ rev	GTAACAGACGGAGCCGCTTTAGG
24	mCherry_ BamHI	CTATGGATCCCCACCATGGTGAGCAAGGGCGAG
25	Sv40_LoxPmCh erry_HindIII	TGCCAAGCTTATAACTTCGTATAGCATACATTATAC GAAGTTATCCCGATCCAGACATGA

Left Junction PCR of pattB_HygroR_eGFP targeting at landing pad in Clone 17 – attR highlighted

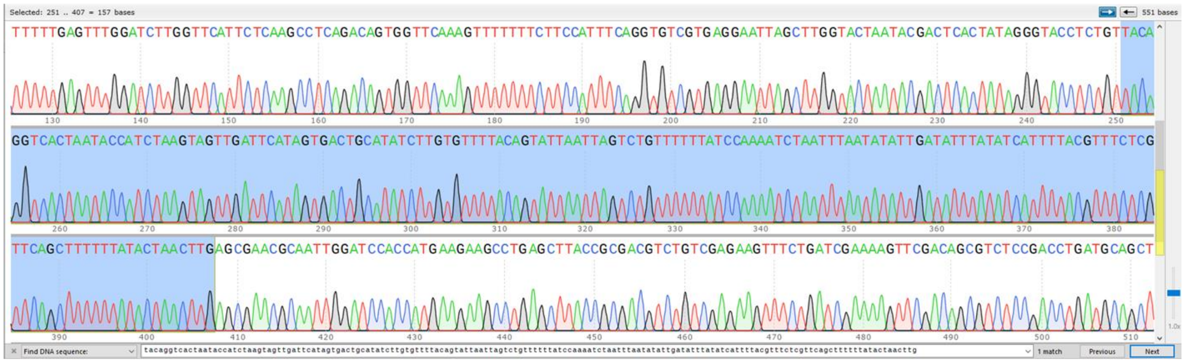

Right Junction PCR of pattB_HygroR_eGFP targeting at landing pad in Clone 17 – attL highlighted

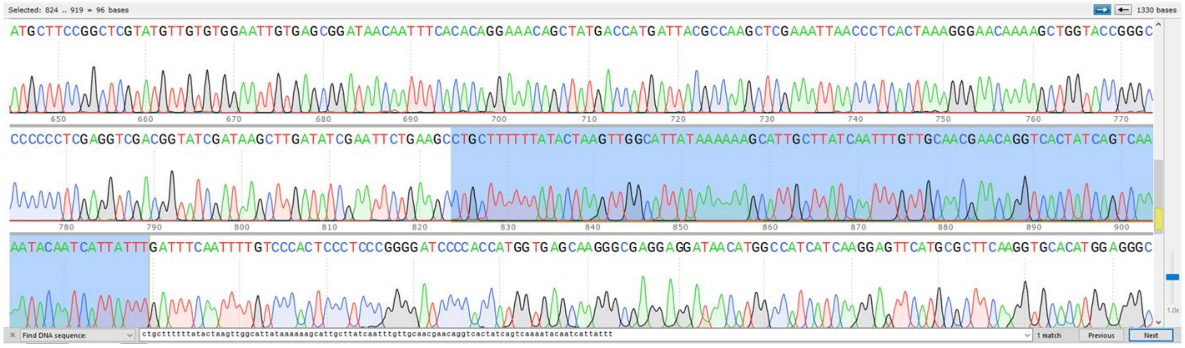

Supplementary figure S1: Sequencing confirmation of the PCR products. Successful targeting of pattB_HygroR_eGFP at landing pad site was tested by the PCR and sequencing to confirm the formation of corresponding attR and attL sites.

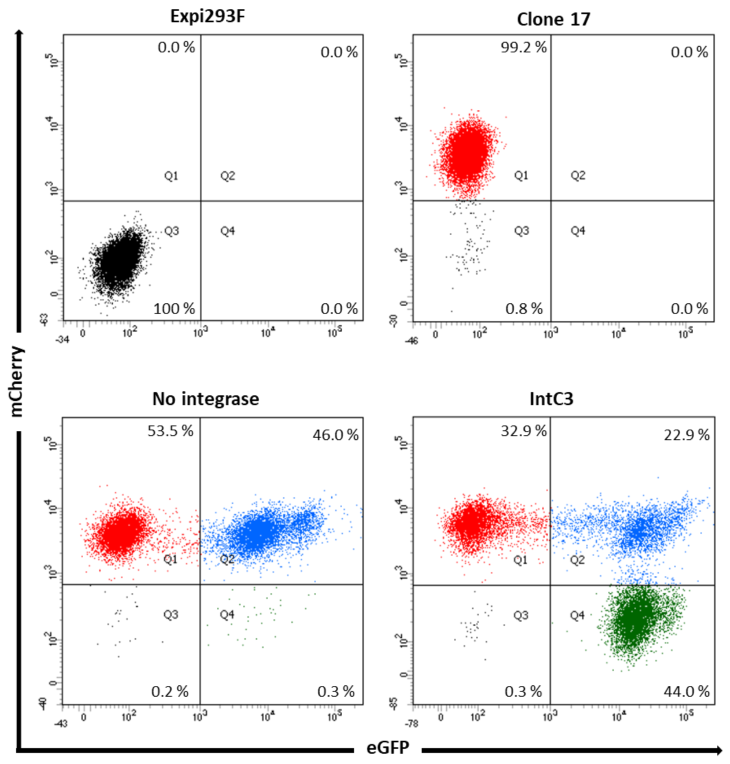

Supplementary figure S2: Targeting efficiency at landing pad in clone 17. Clone 17 was transfected with p*attB*_HygroR_eGFP in absence/presence of intC3 to estimate the number of positively targeted cells after antibiotic selection by flow cytometric analysis. Dot plot representing mCherry negative and eGFP negative Expi293F cells, mCherry positive and eGFP negative clone 17, mCherry positive or double positive cells targeted without intC3 and appearance of single eGFP positive cells upon targeting with intC3.

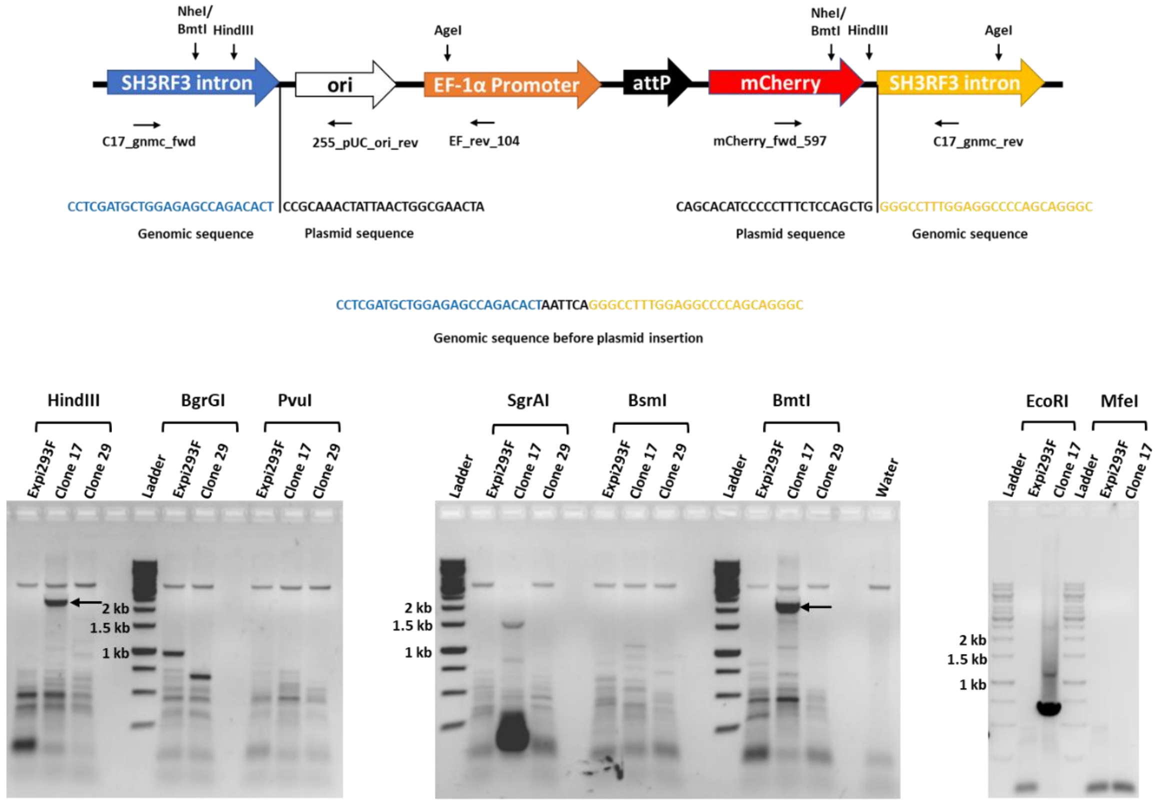

Supplementary figure S3: Detection of specific site of landing pad insertion by inverse PCR. Schematic representation of the site of landing pad insertion in Chromosome 2 with the location of restriction enzymes and primers. Inverse PCR and nested PCR were performed after HindIII, PvuI, SgrAI, BsmI, BmtI, EcoRI and MfeI digestion of clone 17 genomic DNA with EF_rev_104 and mCherry_fwd_597 primers and nested PCR product was resolved on agarose gel. DNA band marked with an arrow was excised and extracted DNA was sequenced to identify the site of landing pad insertion in the genome.

Left Junction PCR of pEF_attP_mCherry insertion in SH3RF3 intron

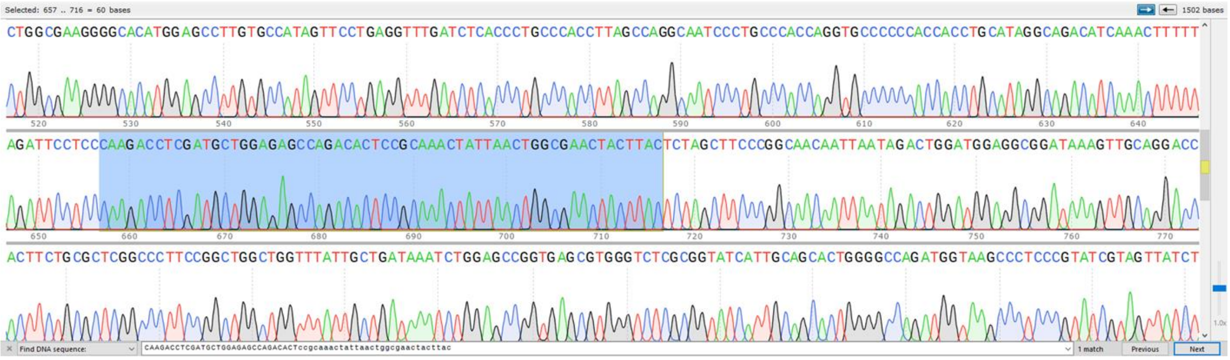

Right Junction PCR of pEF_attP_mCherry insertion in SH3RF3 intron

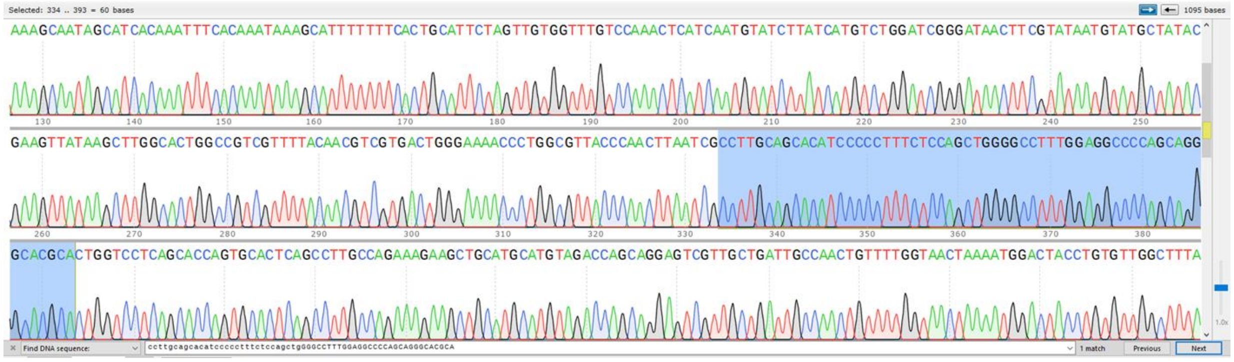

Supplementary figure S4: Sequencing confirmation of the PCR products. Genomic location of the landing pad after insertion was confirmed by PCR and sequencing. Thirty nucleotides upstream and downstream of both left and right junction formed after insertion are highlighted in the figure.

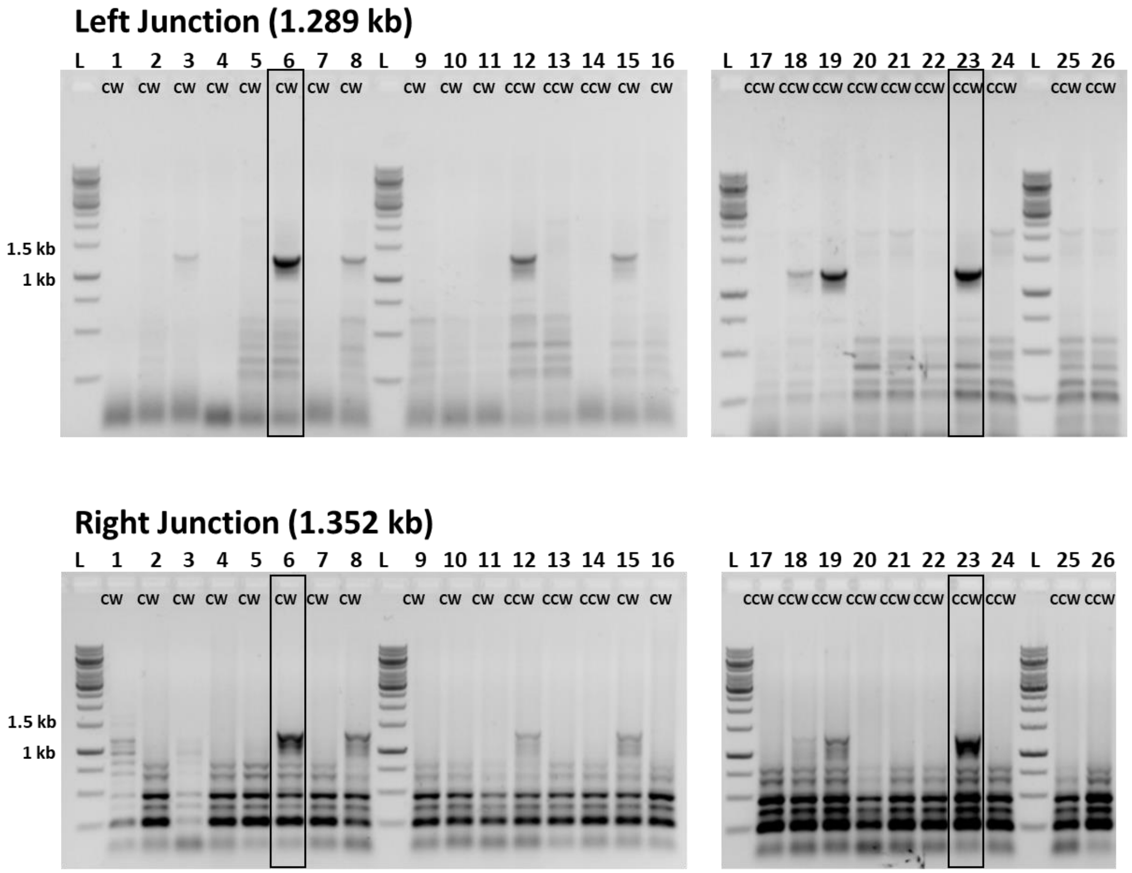

Supplementary figure S5: Screening of IgG transgenes targeted colonies of clone 17. Colonies were isolated after transfection and antibiotic selection. Left junction PCR was performed with genomic DNA from different clones using 39_EF_fwd and Amp_rev_498 and expected 1.289 kb product was obtained. Similarly, right junction PCR was performed using 231_Puro_rev and 66_mCherry_rev to get 1.101 kb product. Subclones obtained from dilution of clones 6 and 23, marked by black border, were further used for protein expression. Ladder denotes 1 kb DNA ladder.

Left Junction PCR of pattB_HygroR_PD1 targeting at landing pad in Clone 17 – attP highlighted

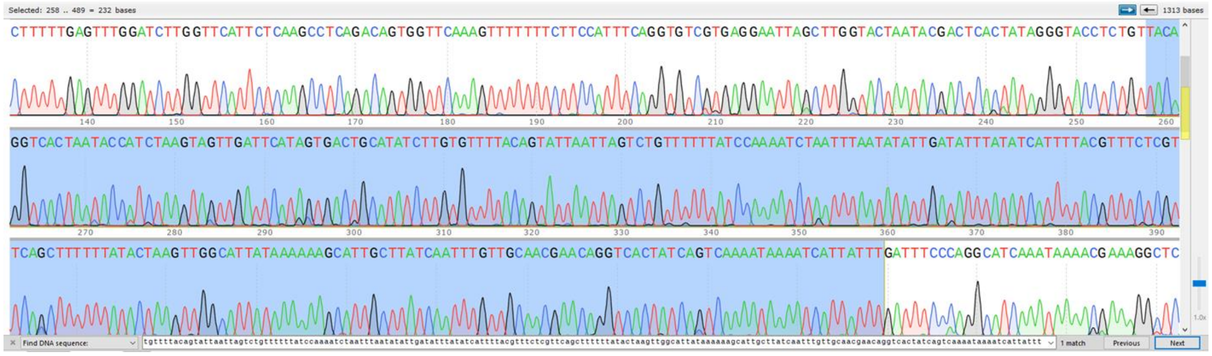

Right Junction PCR of pattB_HygroR_PD1 targeting at landing pad in Clone 17 – attL highlighted

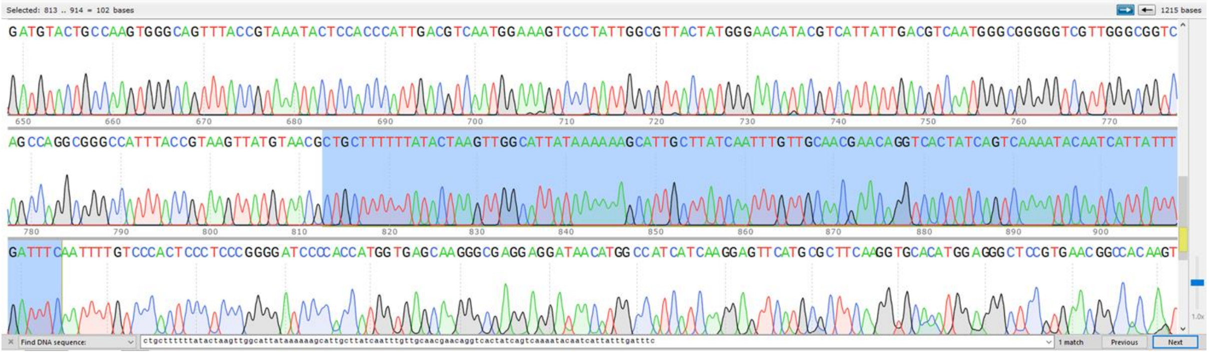

Supplementary figure S1: Sequencing confirmation of the PCR products. Successful targeting of pattB_HygroR_PD1 at landing pad site was tested by the PCR and sequencing to confirm the formation of corresponding attR and attL sites.

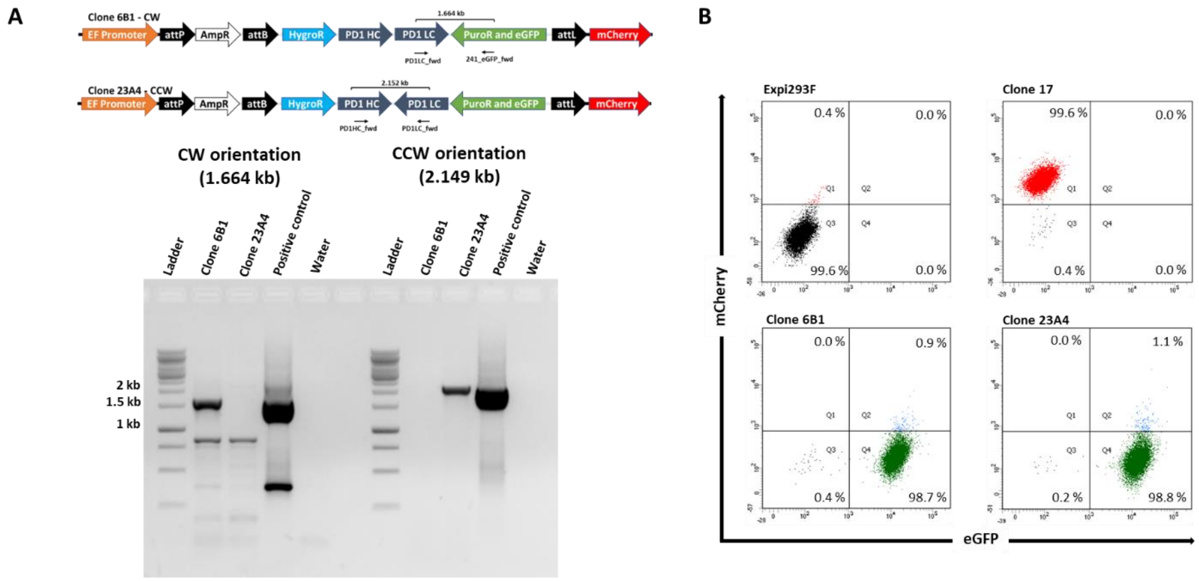

Supplementary figure S7: Confirmation of orientation and purity of IgG transgenes targeted clones. **A.** For CW orientation, PCR was performed with genomic DNA from clone 6B1 and 23A4 using PD1LC_fwd and 241_eGFP_fwd and expected 1.664 kb product was obtained. Similarly, for CCW orientation same PCR was performed with PD1HC_fwd and PD1LC_fwd primers to get 2.152 kb product. **B.** Both clones 6B1 and 23A4 were found to be homogenously single eGFP positive by flow cytometric analysis, lower right quadrant in lower panel.

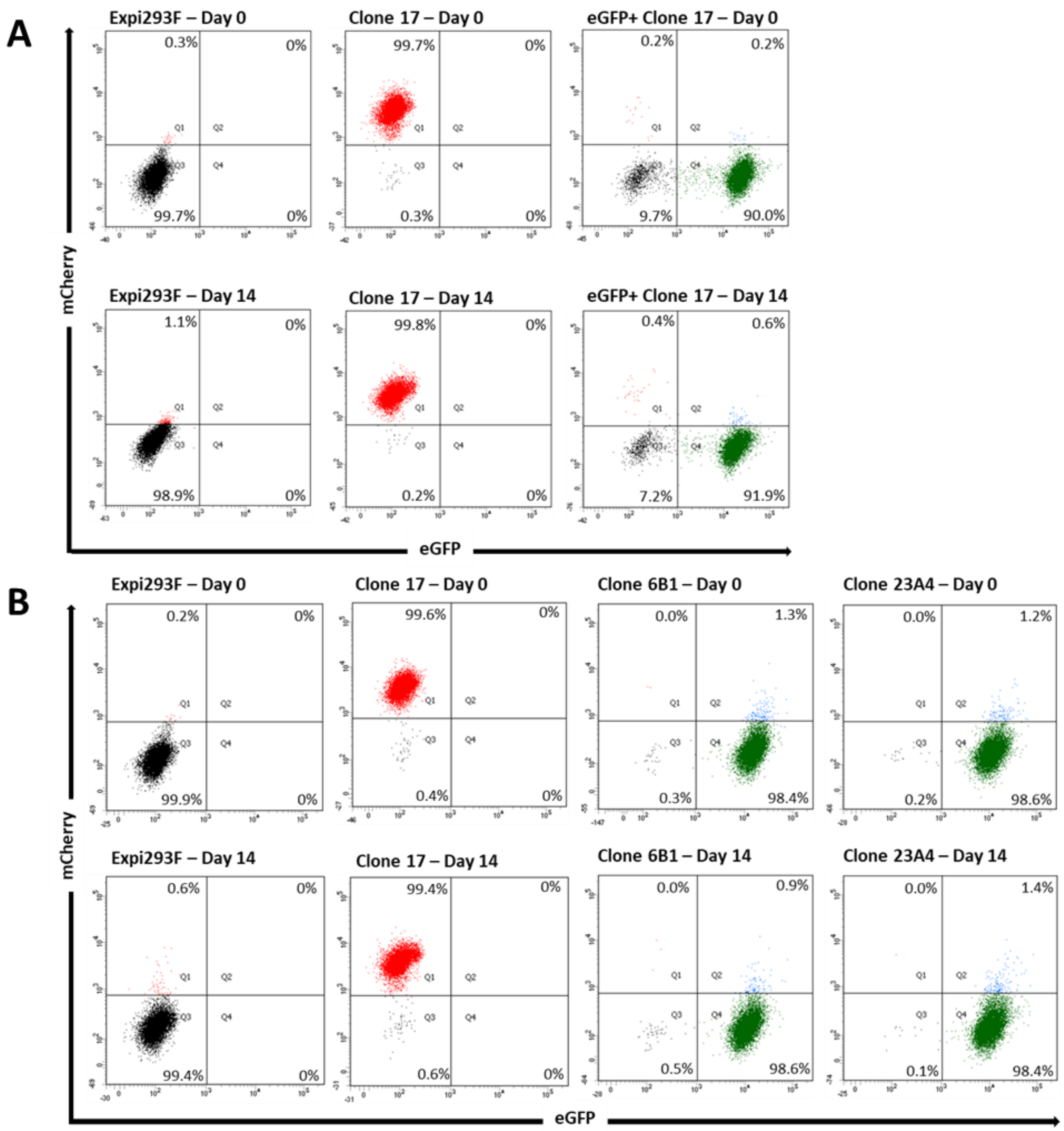

Supplementary figure S8: Stable expression of transgenes targeted at landing pad in clone 17. **A.** Green positive colony, picked after clone 17 transfection with p*attB*_HygroR_eGFP and antibiotic selection, sustained same level of eGFP expression after 14 days of continuous culture as shown by flow cytometric analysis. **B.** Similarly, IgG transgene targeted clone 6B1 and 23A4 maintained similar level of homogenous eGFP expression after 14 days of culture.

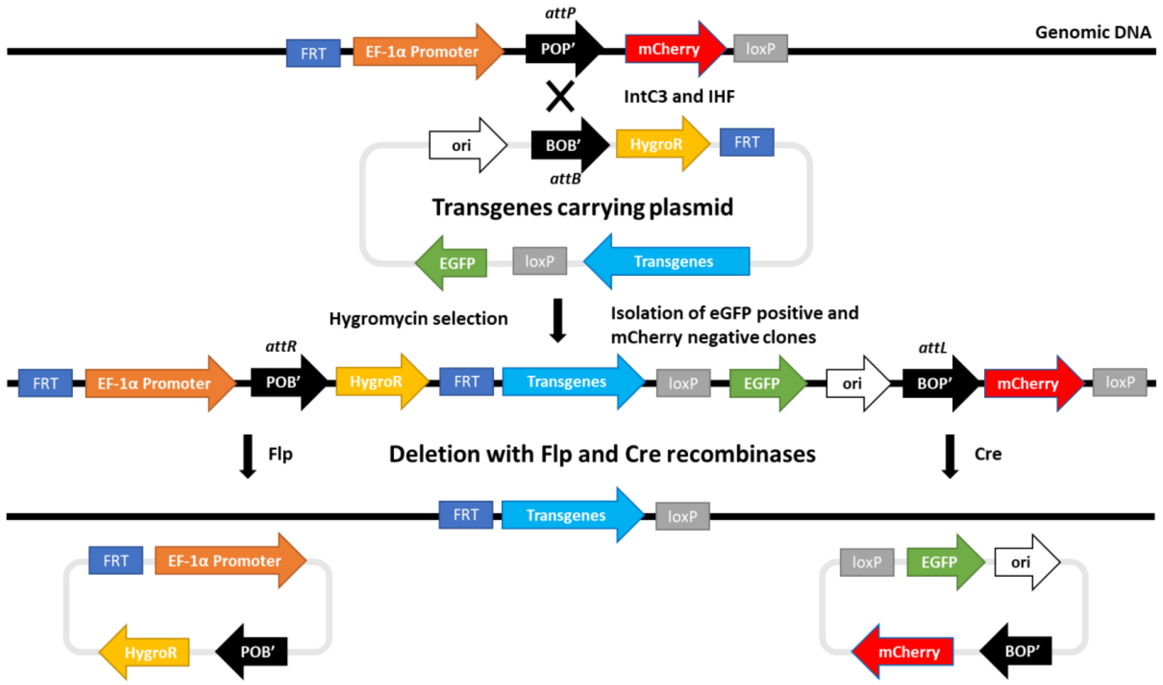

Supplementary figure S9: Removal of unwanted DNA sequences from targeted clones. Schematic representation showing application of Flp and Cre recombinases to remove bacterial backbone, fluorescence and antibiotic resistance genes from the genome of targeted clones. With this method, pure cell lines can be generated with only transgenes.
